# Federated learning in cloud-edge collaborative architecture: key technologies, applications and challenges

**DOI:** 10.1186/s13677-022-00377-4

**Published:** 2022-12-15

**Authors:** Guanming Bao, Ping Guo

**Affiliations:** School of Computer Science, Nanjing University of Information Science and Technology, Ningliu Road, 210044 Nanjing, China

**Keywords:** Federated learning, Cloud-edge collaborative computing

## Abstract

In recent years, with the rapid growth of edge data, the novel cloud-edge collaborative architecture has been proposed to compensate for the lack of data processing power of traditional cloud computing. On the other hand, on account of the increasing demand of the public for data privacy, federated learning has been proposed to compensate for the lack of security of traditional centralized machine learning. Deploying federated learning in cloud-edge collaborative architecture is widely considered to be a promising cyber infrastructure in the future. Although each cloud-edge collaboration and federated learning is hot research topic respectively at present, the discussion of deploying federated learning in cloud-edge collaborative architecture is still in its infancy and little research has been conducted. This article aims to fill the gap by providing a detailed description of the critical technologies, challenges, and applications of deploying federated learning in cloud-edge collaborative architecture, and providing guidance on future research directions.

## Introduction

In recent years, with the advancement of manufacturing, the number of IoT devices such as smartphones, smartwatches, and tablets has grown exponentially. According to IoT analytics, dedicated to IoT market research, the number of IoT devices worldwide has reached 12.3 billion in 2021, and it is still increasing rapidly. At the same time, with the advancement of sensor and communication technology, the ability of IoT devices to collect behavioral data of users (often involving their privacy) is getting stronger, making the scale of the edge data rise massively. The maturity of machine learning (ML) and deep learning (DL) has made the huge amount of edge data of great value [1], such as spelling prediction [2] and personalized recommendation [3]. More applications are landing in many other industries, such as intrusion detection in the Industrial Internet of Things (IIoT) [4], AI diagnosis in the healthcare industry [5], and traffic analysis in smart transportation [6].

However, due to the increasing demand for information security and privacy, many privacy protection laws have been enacted in recent years, such as GDPR [7] and the Consumer Privacy Bill of Rights in the U.S. [8], which makes the traditional centralized data processing methods no longer suitable and gradually popularizes distributed machine learning methods. In cloud-based centralized ML, the data involved in training are pooled into a data center where ML training is performed, and the model parameters are sent back to each client after the training is completed. However, since the raw data often involves private information of users, there is a significant risk of privacy leakage when gathering the data into a central data pool [9]. In addition, due to competing interests, data from large enterprises is often not interoperable, resulting in massive “data islands” at the edge, while machine learning is a technology based on a large amount of data, which makes the advancement of ML seriously hampered [10]. In response to the issues, federated learning (FL), a distributed machine learning method, has been proposed in recent years to solve the problem of “data islands”, which allows the raw data involved in training to be kept on the user side, and then the local model parameters are sent to a model manager without sharing the raw data. The model manager then performs the local model aggregation, and start another round of training. Thus, FL is considered to be a promising machine learning method for allowing multiple data pools for confidential training.

In addition to the shift of central ML to distributed FL, conventional centralized computing architecture is also shifting to be distributed. Cloud computing enables synchronization of data from multiple ends and improve IoT devices by providing data storage and management, together with fast computing services in the cloud [11], which was once considered as the future of the information age [12]. However, various reasons make cloud computing unable to provide satisfactory services in current IoT. In addition to the security risk of collecting data in a central data pool, the cloud computing model suffers from the following issues:

1. *Computation challenges* Although the computing power of the cloud centers is increasing yearly, the rate of their increase is far from the growth rate of the scale of edge data to be processed.
2. *Communication challenges* In cloud computing, clients need to communicate with remote cloud servers, which are geographically distant and need to build long communication links, resulting in extremely inefficient communication and network congestion. What’s more, the communication delays cannot well support many current real-time demanding applications, such as driverless cars [13]. In addition, in ML for improving the quality of service (QoS), long-distance communication consumes much device power, which degrades QoS.

Due to these issues, centralized computing model is shifting to distributed computing model, where the cloud-edge collaborative model is widely considered to be a promising computing architecture for the future [14]. Edge computing (EC) processes data anywhere on the path from where the data is generated to the cloud computing center, which makes the communication of clients more efficient and obtains much less communication latency to support real-time demanding applications, and upward EC can share the huge pressure of the cloud computing centers, which is a good solution to the realistic problems we described above, therefore, EC has been carefully studied [15–17].

Both federated learning and cloud-edge collaborative computing architectures are based on distributed strategies, and how to reasonably deploy federated learning in the cloud-edge collaborative architecture is the concern of this article. We believe that federated learning based on cloud-edge collaborative architecture is the key infrastructure of future web services, which is recognized by many peers [18–20], and further in-depth research on it is crucial for the development of information science.

Li et al. [21] and Yao et al. [22] provided detailed reviews about cloud-edge collaborative architecture, respectively emphasized detailed collaborative techniques and the collaborative learning mechanisms adapting to the cloud-edge collaborative architecture including pretraining models, graph neural networks and reinforce learning, while they have no discussion upon the promising decentralized federated learning in the collaborative architecture. There are much research aimed at discussing federated learning in edge computing environment [19, 23], however they emphasized EC-enabled techniques and omitted the collaboration among the system entities. In this paper, we discussed FL in the cloud-edge collaborative architecture, our contributions are as follows:

- Introduces the collaborative learning mechanisms for cloud-edge collaborative architecture.
- Identifies the key technologies and challenges for deploying federated learning in cloud-cloud collaborative architecture.
- Presents promising applications of federated learning based on cloud-edge collaborative architecture.
- Presents future research directions for federated learning based on cloud-edge collaborative architecture.

## Cloud-edge collaborative architecture

### Edge computing

Edge computing (EC) is an emerging computing model that considers leveraging computation resources on the edge of the network. The model consists of three layers: cloud computing center, edge servers, and IoT devices, where “edge” means any computation and communication resources between the path from the raw data to the cloud cloud servers [24]. Edge computing transfers part of the computation tasks from the cloud to the edge servers, which improves communication efficiency that is significant in Iot and satisfy real-time requirements [25]. It is worth noting that EC will not replace cloud computing but assists and expands it, which is still the primary and fundamental computing paradigm. The two are complementary. In recent years, the energy consumption generated by cloud computing is also increasing year by year and some time-sensitive applications can not be well supported by cloud computing for the unsatisfied latency. Conventional cloud computing needs to be modified and EC is promising in some emerging applications such as video surveillance, smart cities, and intelligent transportation, but EC also needs to consider the following challenges:

- *Program Migration*: In edge computing architecture, edge nodes often have different Operating Systems (OS), making some code on edge devices run incorrectly or fail to run when transferred to edge servers.
- *Security*: On one hand, the resources of edge devices are insufficient to support large scale traditional privacy methods, e.g. Homomorphic Encryption (HE). On the other hand, some edge nodes and clients may be malicious attackers due to loose management, which may corrupt the accuracy of the joint model.
- *Service Continuity*: Usually, edge devices are connected to the edge server that is geographically close to it. However, in some scenarios, such as the Vehicular Networks, edge devices tend to move to a place that is close to another edge server and connect with the new one. It is a challenge to keep the services continuous in the dynamic process.

### Cloud-edge collaboration

**Fig. 1:**
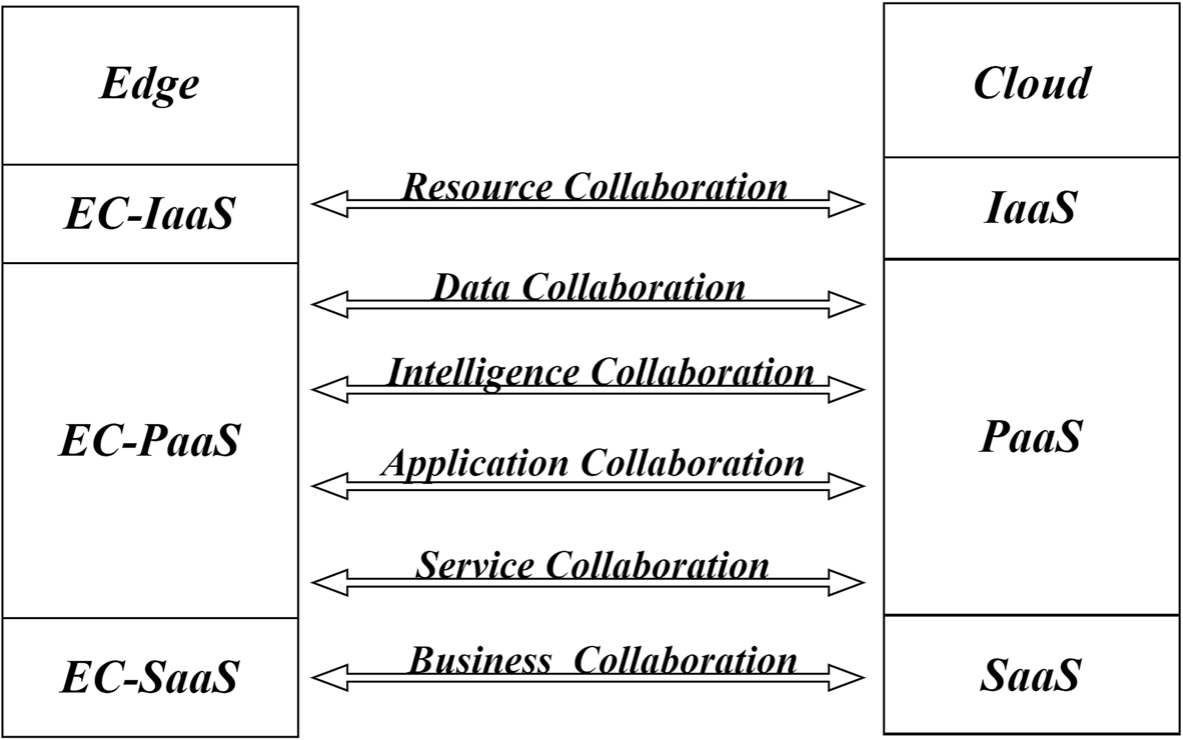
The cloud-edge collaborative architecture

Cloud-edge collaboration refers to the novel computing architecture where cloud servers and edge servers cooperate with each other, jointly providing computing, storage, and other services. It is widely considered to be a promising computing paradigm for the future [26]. In the architecture, Edge Computing (EC) mainly processes data with high real-time requirements [27]. Due to the data in IoT is usually not disposable, the pre-processed data still needs to be gathered from the edge servers to the central cloud servers. Cloud computing mainly processes non-real-time and long-period data and conducts management of edge applications, providing services such as data backup and big data analysis. The cloud-edge collaborative architecture is shown in Fig. 1.

Cloud-edge collaboration involves the collaboration of IaaS, PaaS, and SaaS between the edge and the cloud. In general, IaaS, PaaS, and SaaS are three basic models of cloud and edge computing, or three basic services provided by cloud and edge computing providers. The specific meanings of IaaS, PaaS, and SaaS are as follows:

- *IaaS* Infrastructure as a Service. It refers to the provisioning of infrastructure services that are originally deployed locally, which includes services such as networking, computing, and storage hardware, as well as virtual machines. At the same time, IaaS providers also give a number of accompanying infrastructure services at the same time, including real-time billing, monitoring, log acquisition, firewall and security protocols, backups and replies, etc. IaaS provides great convenience to various organizations because when organizations need to develop a new product, they can not bother to construct a specific infrastructure, but directly purchase or rent the corresponding one from the IaaS provider.
- *PaaS* Platform as a Service. It is based on IaaS for that PaaS not only includes the infrastructure hardware facilities but also provides infrastructure-based software services including operating systems, middleware such as databases, etc. Other PaaS services are application design and development, application testing and deployment, web service integration, information security, and database integration.
- *SaaS* Software as a Service. It is a software distribution model where the provider hosts applications and makes them available to end users over the Internet. Unlike IaaS and PaaS, SaaS products are frequently marketed to both B2B and B2C users. Users do not need to care about setting up the working environment they need, including the installation of applications and system software, which are all contained in SaaS.

The upper layer of cloud-edge collaboration involves many aspects of collaboration, including resource collaboration, application collaboration, data collaboration, intelligence collaboration, service collaboration, and business collaboration, and the lower layer relies on three basic service models of cloud and edge, namely IaaS, PaaS, and SaaS, where the resource collaboration of virtual resources such as computing and network relies on the collaboration between edge IaaS model and cloud IaaS model. Business collaboration, data collaboration, intelligence collaboration, and application collaboration rely on the collaboration between edge PaaS and cloud PaaS. And service collaboration relies on the collaboration between the edge SaaS model and the cloud SaaS model. In this paper, we aim to explore the application and deployment of federated learning in the six types of collaboration in the upper layer of cloud-edge collaboration. What the above six collaboration means is demonstrated as follows:

- *Resource Collaboration* Similar to cloud servers, edge servers are equipped with a relatively small amount of virtual resources such as computation and network. Edge servers have local resource management policies to allocate precious virtual resources. At the same time, the cloud server stands in a global perspective to observe the overall situation of some applications and schedules as well as manages the virtual resources for the edge servers that are distributed geographically adjacent to each other. Resource collaboration can provide better services to end-users. Computation offloading is achieved by offloading the end-user’s local compute tasks to the cloud or edge servers, and the offloading decision process is the means of resource collaboration. Considering other resources like energy, the architecture can provide the smallest possible service latency while minimizing energy consumption [28].
- *Data Collaboration* The job of the edge server is to perform the initial collection and pre-processing of data generated close to the user side, which often involves the user’s privacy, and then hand over this simply processed data to the cloud, which takes a global view of the extensive data for long-term observation and processing.
- *Intelligence Collaboration* The edge server performs simple model inference on the collected edge data, and the cloud is responsible for aggregating the inference models from the edge and performing complex centralized training, and then delegating the final models to the edge servers, which involves typical machine learning methods such as deep learning models and techniques such as model splitting and model compression techniques.
- *Application Collaboration* PaaS services for edge servers enable most edge nodes to have a mainstream application deployment and runtime environment, which schedule and manage the operation of multiple processes locally. Cloud PaaS manages and schedules the processes of multiple edge nodes.
- *Service Collaboration* Due to the law as well as the number of users and other factors, the service level of application products often varies in different regions, and service collaboration is the key technology to achieve flexible service distribution. Edge SaaS submits to the service distribution strategy of cloud SaaS to realize SaaS services, and cloud SaaS needs to propose a service distribution strategy to edge SaaS in addition to providing cloud SaaS services.
- *Business Collaboration* Edge servers provide modular, micro-services-based application instances and the cloud provides the ability to orchestrate business according to customer needs.

The design and deployment of cloud-edge collaborative architecture are still in their infancy, where solving resource collaboration, data collaboration, intelligence collaboration, Application collaboration, business collaboration, and service collaboration are six significant issues [21]. Currently, researchers have tried to apply cloud-edge collaboration to possible fields, and the main application areas are concentrated in content delivery network (CDN), Industrial Internet of Things (IIoT), Energy, Smart Home, Intelligent Transportation, Secure Monitoring, Agriculture, Healthcare, and Cloud Games [29].

### P2P collaborative architecture

Apart from the client-server collaboration between the edge servers and the cloud server, edge nodes can also collaborate with each other and comprise Peer-to-Peer (P2P) collaborative architecture, further improving the performance of the architecture, which needs to refer to the P2P network. P2P network models can be classified into centralized P2P and distributed P2P [30]. In the centralized P2P model, one or more cloud servers are deployed to record the dynamic status of the distribution of resources among the peers, while distributed P2P is the pure network of peers, each of which has equal privilege. Therefore, we are supposed to refer to centralized P2P in this paper, where each edge node can not only collaborate with the cloud servers but also with other nearby edge nodes. Introducing decentralized P2P networking among the edge servers, the collaborative architecture can be more robust for that P2P mitigates the single point of failure in the naive cloud-edge architecture [31]. The introduction of P2P can make the architecture more flexible and robust when the edge nodes are mobile [32]. Therefore the application of P2P network in cloud-collaborative architecture is promising, Tang et al. [33] used P2P network to realize offloading in MEC and obtain efficient and feasible performance. Currently, the application of P2P in cloud-edge collaborative architecture is still in its infancy, most research ignores the direct collaboration among the edge nodes. In this paper, we touch upon some applications of P2P networks in cloud-edge collaborative architecture.

## Federated learning

In order to keep the training data in clients, Federated Learning (FL) is proposed, which is a decentralized ML framework. A joint model resides in the cloud server, and the data for training are distributed in different devices. It is worth noting that the involved data from different sources are usually not independent and identically distributed (Non-iid). Mcmahan et al. [2] proposed the conception of FL and the first FL algorithm, i.e., Federated Averaging (FedAvg). FedAvg mainly includes the following three steps:

1. Initialization: The global and local model parameters are initialized to the same value.
2. Local updates: A certain number of clients are selected randomly. Each selected clients perform gradient descents on their local data.
3. Global update: Global model parameters are updated as a weighted average of all local model updates.

After iterating 2 and 3, a better model can be obtained. In FedAvg, only the model parameters are involved in communication, and raw data transmission is avoided.

A widely received taxonomy of federated learning is: (i) Horizontal Federated Learning (HFL), (ii) Vertical Federated Learning (VFL), and (iii) Federated Transfer Learning (FTL) [34]. HFL is the union of samples, which is applicable when most features while few samples overlap e.g. sharing diagnosis data between hospitals in different regions for training a more robust model to make accurate diagnoses. VFL is suitable when there are many samples overlapping and few features overlapping, e.g., banks and Internet companies sharing data to model client credit for risk control. FTL applies when both the samples and the features overlap little, e.g., start-up financial companies can get data from open financial data to learn and improve their service capabilities.

While federated learning is safer compared with the conventional centralized ML and can efficiently process data silos, it can not always work. The main challenges in FL are statistical heterogeneity, system heterogeneity, and model heterogeneity [35]. Statistical heterogeneity is the case when the available local data can not represent the overall distribution System heterogeneity refers to clients participating in FL often having distinct hardware conditions, such as network, battery, computing ability, and storage capacity. Some devices may be unable to return the local updates in time due to their constrained resources, and most FL setting is synchronous, which may prolong the convergence. Model heterogeneity usually occurs in business-to-business (B2B) FL, where different clients may have different requirements for the model due to their different expectations, but in FL only one global model is provided for each client, and having good prediction performance for all the clients is a challenge. Additionally, most of the previous FL algorithms assume that clients are honest, which poses a security risk. The problems mentioned above make the model trained by FL may not outperform the local model for some clients, which makes the client reluctant to take part in the training. In view of the above situation, the evolution of federated learning is significant for IoT applications, which are highly dependent on addressing challenges, e.g., statistical heterogeneity, system heterogeneity, model heterogeneity and secure management, where statistical heterogeneity is more pervasive, which we introduce as follows:

### Statistical heterogeneity

Statistical heterogeneity is the prominent challenge federated learning is confronted with. The available local data can not represent the overall distribution [36]. We assume a training task including features *x* and labels *y*. The union local data distribution of client *i* can be described as *P*(*x*, *y*). There are many forms of Non-iiD in the different learning tasks:

- *Feature distribution skew* (covariate shift): the marginal distribution *P*(*x*) varies between different clients. Such as, the same verbal content spoken out by different people can be distinct in terms of timbre and tone in speech recognition [37].
- *Label distribution skew* (prior probability shift): the marginal distribution *P*(*y*) differs across clients. In different usage environments, the same feature can generate multiple labels.
- *Same label corresponding to different features* (concept drift): the conditional distribution *P*(*x*|*y*) varies. Similar features in certain clients at a particular time or in various places can correspond to multiple labels.
- *Same feature corresponding to different labels* (concept shift): the conditional distribution *P*(*y*|*x*) differs, e.g., in Gboard, the same words “I want to” will be predicted to “go sleeping” for user A and “have something to drink” for user B.
- *Quantity skew or unbalancedness*: Participants in FL may be as small as a smartwatch to as large as a hospital, where the number of data varies considerably.

Mcmahan et al. [2] demonstrated that FedAvg works well on Non-IID data, but much research [38–40] found that the derived model precision will degrade remarkably on Non-IID data. To measure Non-iid, Li et al. [36] proposed a way to evaluate the extent of Non-IID with the sum of the global objective function and local objective function, which is proved to be a positive correlation with the extent of Non-IID. Much research had been done to optimize FL from the aspect of statistical heterogeneity [41, 42] and achieve excellent results.

### Edge federated learning

**Fig. 2:**
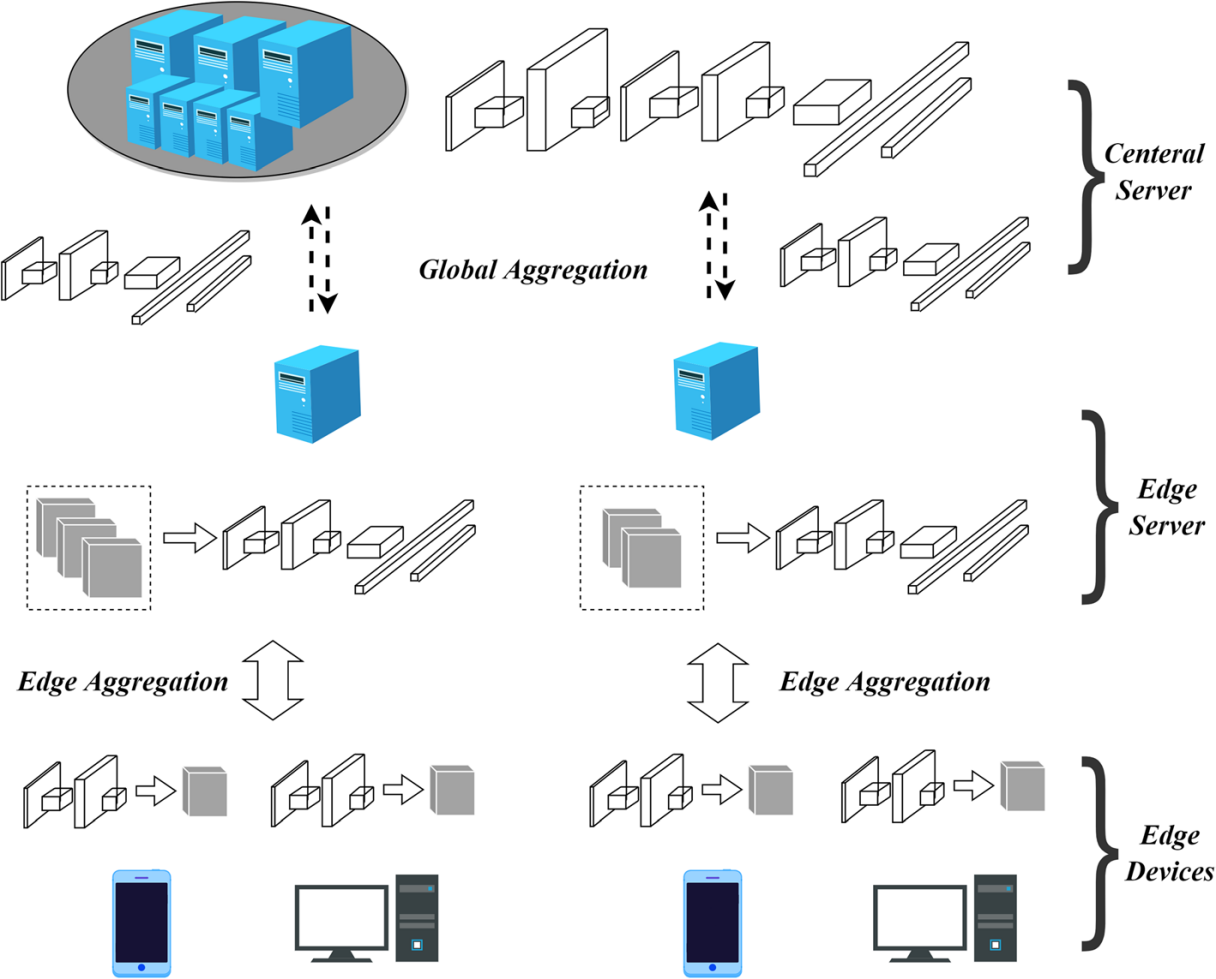
The framework of edge federated learning

In the cloud-edge collaborative architecture, FL can be optimized by the cloud-edge collaboration and in this paper we call it edge federated learning. However, the complex environment and heterogeneity of IoT bring significant challenges to deploying edge federated learning [35]. As shown in Fig. 2, we introduce a popular edge FL framework. The framework consists of three layers: the central cloud server, edge servers, and edge devices, which are the three essential elements in the edge computing model.

- *Edge devices*: Edge devices are usually portable devices distributed on the edge of the network, e.g., smartphones, smartwatches, and tablets. They are usually equipped with relatively limited computing and storage resources in terms of portability, which means that they are not good at performing large-scale computing tasks. They collect and store much user-generated data, and the data often contains users’ privacy.
- *Edge servers*: Edge servers are typically positioned in proximity to edge devices, the communication link is short, and the bandwidth resources are relatively abundant, so the communication is fast and efficient between the edge servers and the edge devices. Edge servers usually have much richer computing and storage resources and a more stable power supply than edge devices. They are promising to share the computing tasks of edge devices and expand their capabilities.
- *Cloud server*: Cloud computing centers are geographically far from edge devices and edge servers, with slower communications and limited bandwidth. The operators provide the central servers with vast storage resources and mighty computing power, which are suitable for large scale computing tasks.

Different from traditional FL [2], in edge federated learning, the cloud server first distributes an original global model to the edge servers, and edge devices request the assigned edge server to download the initial model parameters. Similarly, edge federated learning allows the local model outputs on the edge devices to be aggregated on the edge server first [19], after iterations, global aggregation is performed between the edge servers and the central server. The central server then distributes the global model parameters to the edge devices through edge servers when the model converges to the set accuracy.

### Types of federation

There are mainly two FL settings in real-world applications [20], i.e., cross-device FL and cross-silo FL. They apply to different application scenarios respectively. Cross-device FL is usually applied to the learning between a large number of IoT devices to improve QoS, e.g., spelling prediction. Cross-silo FL is often used in training between large institutions to maximize prediction accuracy, e.g., collaborative training of disease prediction models with higher accuracy among multiple medical institutions. It is worth noting that this taxonomy does not cover all application scenarios of FL. We list the differences and commonalities as follows:

#### Differences

We compared the two different FL settings, as shown in Table 1. The significant feature of cross-device FL is the large-scale clients, which brings challenges such as communication bottlenecks and high concurrency. Cross-silo FL involves much fewer clients where a large number of samples are reserved by each client, requiring the clients to perform much more computation.

**Table 1:** Differences between cross-device FL and cross-silo FL

Aspects	Cross-silo FL	Cross-device FL
Setting	Clients are usually large institutions. Each client reserves a large number of training samples and has sufficient training resources.	Clients are usually portable devices and lack training resources.
Data availability	Clients are almost always available	Only some clients can be used for training at certain time periods.
Federation scale	The number of clients is usually less than 100.	The amount of the client is about $10^{10}$.
Primary bottleneck	Communication and computation	Communication
Addressability	Each client is assigned an identity.	Customers usually cannot be directly indexed.
Client reliability	Usually few errors.	High error rate.
Data partition axis	Horizontal or vertical.	Horizontal.

#### Commonalities

Although there are many differences between cross-device FL and cross-silo FL, they all originate from conventional FL and there are also many similarities between them. We demonstrate these similarities in architecture and challenges as follows:

*Architecture*

- They all store the data locally and each client cannot obtain the data of other clients.
- Different from the network topology of peer-to-peer communication, both of the two FL settings are star network topologies. The center is a training manager, and the nodes are clients.

*Challenges*

- They are all confronted with privacy and security challenges. Privacy leakage in cross-device FL may lead to users’ personal data being illegally used and data leakage in cross-silo FL may cause an inestimable economic loss to the institutions.
- The training resources consumed by the two settings are all huge. The communication consumed in cross-device FL is mainly due to the huge number of clients. Cross-silo FL needs to process massive data samples, requiring much computation.
- Both of them need to solve statistical heterogeneity, system heterogeneity, and model heterogeneity.

### Cross-device federated learning based on cloud-edge collaboration

Compared with the traditional cross-device FL, cross-device FL can be further improved in cloud-edge collaborative architecture. By deploying edge servers, edge networks can protect edge traffic, and the edge servers under attack can be withdrawn from training, where attacks on a local training governed by an edge node will not affect other local training [43]. In addition to the security enhancement, local computation tasks performed by clients can be offloaded to the edge servers in a secure manner [44], and this can provide low-latency computation services to mobile devices with sufficient communication bandwidth between clients and the edge servers, thus reducing the computation of clients involved in the training.

### Cross-silo federated learning based on cloud-edge collaboration

In the cloud-edge collaborative architecture, cross-silo FL has more possibilities. In cross-silo FL, the local dataset in each client is more suitable to be seen as a separate learning task rather than the set of data fragments and one of the most important challenges is that when the data distribution between silos is significant, there will be serious Non-IID issues. In the traditional cloud computing architecture, meta-learning and transfer learning [45] are often used to solve Non-iid. Cloud-edge collaborative architecture provides a novel method of solving Non-iiD, i.e., Hierarchical Federated Learning based on clustering. The easiest way to design a cluster-based method is to divide clients according to data distribution and put clients with similar data distributions in the same learning task, and manage through the edge servers [46], Beiggs et al. [47] confirmed that this method is effective to solving Non-iiD issues.

### Split learning

Split learning (SL) also called split neural networks (splitNN) was first introduced by MIT Labs. SL is a distributed and private deep learning technique, aiming to train a deep neural network over multiple data clients and one central server. SL can satisfy the following requirements: (i)data clients do not want their local sensitive data seen by other clients or the central server (ii) The central servers can keep some net parameters for inference (iii) The central server can control the overall architecture of the training. In SL, the novelty is that the deep neural network is split into multiple sections, and each of them is trained on a different data client. Every client trains one part of the deep neural network to the same layer, that is called cut layer, and then the outputs of the cut layer on the current client are transferred to the other client rather than the raw sensitive data. By orderly relaying the forward propagation, the rest of the forward propagation can be completed. Due to the relay-based training process, split learning is relatively slow than some other distributed machine learning methods, e.g. federated learning. After forward propagation, the gradients are back propagated from the last layer until the cut layer in a similar fashion, similar to forward propagation, only the gradients at the cut layer on clients are transferred to the central server and the rest of back propagation is completed in the central server [48]. The above process is continued until the splitNN is trained and this process is the simplest configuration for SL. There are many other possible configurations for SL and we introduce other two of them.

- *U-shaped SL* The above simple SL configuration are not private at some cases when the label is sensitive, such as the health situation and the financial status of the data clients. The U-shaped configurations can compensate for the deficiency. At end layers, the deep networks in the central servers are wrapped around and the outputs are transferred to data clients, from which the clients compute the gradients and perform back propagation without exchanging labels.
- *Vertical SL* Similar to VFL we discussed above, vertical split learning (VSL) applies to the scenarios when the data samples overlap a lot while few features overlap. When training a splitNN between two organizations when they have many common clients but they run different businesses, they two firstly train different partial models to the cut layer, then the outputs of the two cut layers are combined and transferred to the central server for the rest of the training. Then iterate the process until convergence.

SL is promising for its huge improvement of computational resource efficiency and the reduced communication costs over other distributed learning techniques like FL, however, SL is slower than FL for its relay-based training process. Therefore, Chandra et al. [48] combined the two popular distributed learning methods and proposed novel architecture combining their advantages. Besides the current distributed learning techniques, more work needs to be done to realize efficient and secure distributed learning.

## Key technologies of applying federated learning into cloud-edge collaborative architecture

**Fig. 3:**
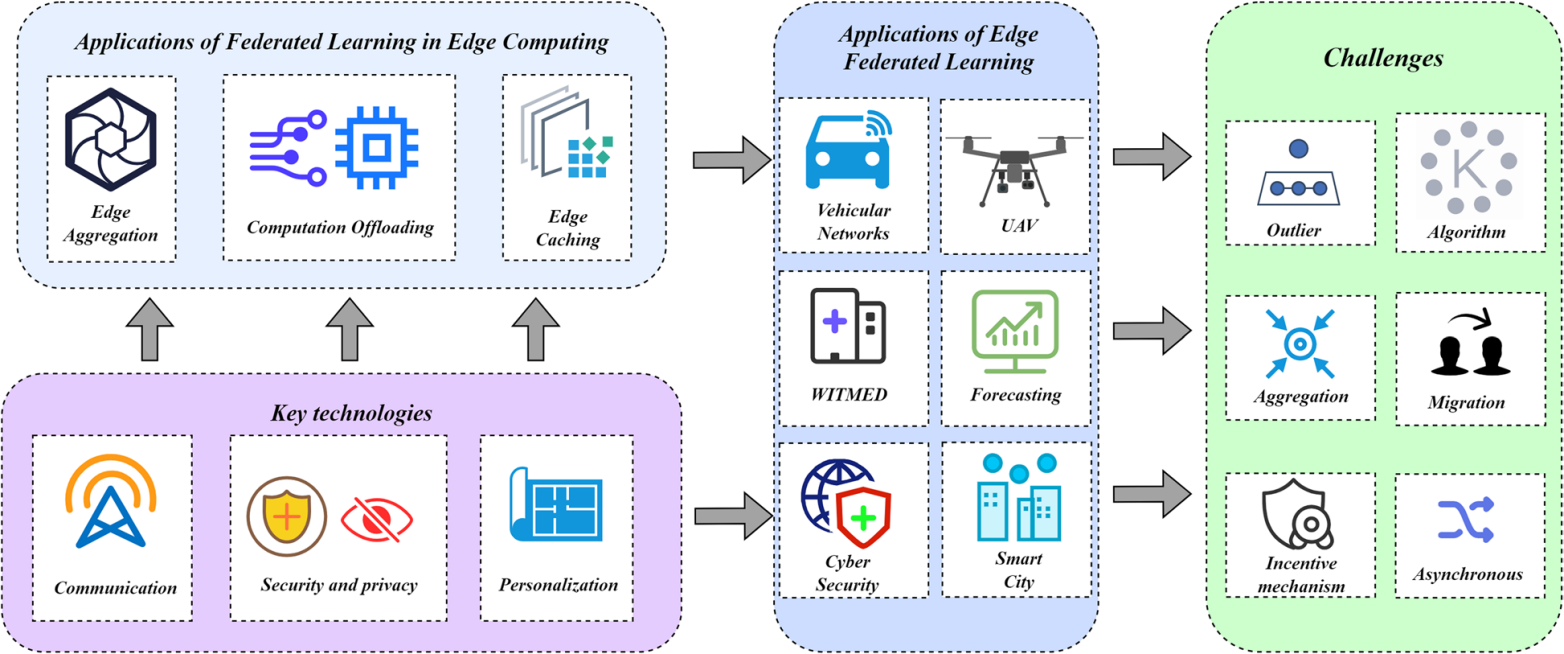
The research architecture of federated learning in cloud-edge collaborative networks

This section focuses on three key technologies for deploying federated learning in the cloud-edge collaborative architecture, i.e., communication, privacy and security, and personalization. In the next two sections we will talk about the applications and challenges respectively, and the research architecture is shown in Fig. 3.

### Communication

In the previous section, we illustrated that communication bottlenecks are a common challenge for both cross-device FL and cross-silo FL. Although clients can communicate more efficiently with edge servers, in FL, clients always need to perform many rounds of communication to make the model converge, and the size of a local model can be on a large scale, which may consume much training cost of the clients and incur unacceptable communication latency, especially in cross-device FL. Therefore, achieving efficient communication is widely considered to be a key technology for deploying federated learning in cloud-edge collaborative architecture. After research, we have compiled three effective ways to achieve efficient communication as follows:

#### End computing

Considering (i) The computing power of mobile devices is increasing and (ii) The data in clients is on relatively small scale, mobile devices are considered to be capable of performing more local computations. Moreover, users tend to place more emphasis on communication resources compared to computational resources, e.g., users tend to participate in FL only if they are connected to WiFi. In view of the above situations, end computing tries to make FL clients perform more local computations, and edge servers perform more edge aggregations before global updates, accelerating the convergence rate of the model, to reduce the overall communication rounds. Mcmahan et al. [2] proposed two ideas for increasing computation: (i) increase the number of gradient descents on edge devices. (ii) increase parallelism to involve more clients in training. However, considering that the increase in computation is limited, the optimal trade-off between computation and communication is a problem to be addressed. In their experiments, simulations based on datasets conforming to the IID distribution showed that (i) increasing the amount of computation on the clients can reduce the number of communication rounds by more than 30 times. (ii) There is a threshold for the reduction of communication rounds by increasing parallelism, and when the threshold is exceeded, the number of communication rounds hardly decreases.

#### Aggregation control

Aggregation control reduces the number of communications by controlling the frequency of aggregation and the number of devices involved. Mills et al. [49] proposed a distributed ADAM optimization to tune FedAvg. To reduce the number of iterations required for convergence, they explored novel compression techniques and proposed a communication-efficient variant of FedAvg, which they claimed could reduce the communication cost to one-sixth of FedAvg. Liu et al. [50] considered deploying federated learning in the vehicular networks and they proposed a new communication protocol, FedCPF. The method allocates part of clients to participate in the communication to avoid major concurrency and limits the communication time in each round, which provides a flexible solution. In [51] and [52], asynchronous aggregation is studied to avoid communication inefficiencies caused by lagging clients.

#### Model compression

In FL based on cloud-edge collaborative architecture, transferring gradient vectors between clients and edge servers is an unavoidable burden, but leveraging some approaches such as quantization, sparse and low-rank approximation to compress the models that clients need to upload, which means uploading partial rather than complete information, can improve the communication efficiency. However, since model compression is a lossy and inaccurate process, it is required to be able to maintain the training quality. Albasyoni et al. [53] investigated the trade-off between the number of bits required to encode the gradient vector and the compression error. They designed two trade-off operators to cope with two different scenarios, and they found that the quality of model training hardly degraded after the compression. Sattler et al. [39] designed a specialized compression method, i.e., Gradient Ternary Compression for FL, which is an extension of TOP-K gradient sparsity and performs well in their four reference FL tasks. We conjecture that model compression methods such as STC are equally effective in cloud-edge collaborative architectures, i.e., model communication between edge-client and cloud-edge can be well improved by introducing appropriate model compression methods, maintaining the training quality of the models and achieving efficient infrastructure services.

### Privacy and security

There are usually two hypotheses in most cloud-based FL frameworks: (i) All devices involved in FL including clients and edge servers are honest. They strictly abide by the requirements of the FL manager. (ii) All clients can not get access to the data from other clients. They simplify the FL system, but the edge servers and clients may not be fully trusted in cloud-edge collaborative architecture, and malicious attackers can easily participate in FL, without considering these two assumptions it is impossible to make FL reliable infrastructure in the cloud-edge collaborative architecture, which may well cause privacy leakage and inability to defend against illegal attack [54], disrupting the social order and bringing negative impact to clients. Therefore, security and privacy are critical technologies when deploying FL in cloud-edge collaborative architecture.

#### Security

FL is designed to protect the confidentiality of the training data in clients, which means the aggregators (edge servers or cloud servers) have no knowledge of how the uploaded vectors are generated, and thus FL is vulnerable to the malicious vectors uploaded. e.g. (i) data-poisoning attack: arranging some malicious clients to participate in FL with much mislabeled data, Tolpegin et al. [55] demonstrated that even a small number of malicious participants can cause great harm to the joint model, and Wang et al. [56] detailed the destructive nature of this attack including the hidden causes. Compared to data-poisoning attack, (ii) model-poisoning attack, is more destructive. Malicious participants can introduce backdoor functionality into the joint model by *model replacement*. Bagdasaryan et al. [57] showed that by tampering with the classifier, the model-poisoning attack can cause fatal damage to FL and the failure cannot be detected by FL managers for most conventional FL aggregators see all the clients as the same.

Two effective defenses against the above two poisoning attacks are proposed in [58]: (i) overall failure: for updates from each participant, the aggregator checks whether the update can improve the performance of the joint model. When the global model performance decreases, the client is flagged as a possible malicious participant, and the aggregator identifies a client as an attacker when multiple rounds of updates are found to degrade the joint model. (ii) client differences: The goal of attackers is usually to make the global model classify a set of highly concentrated mislabeled data samples, so the attackers can also be determined by judiciously comparing the model size of any two clients. When the model updates are large, the client is likely to be an attacker, and after multiple rounds of observation, the aggregator can filter out most attackers.

#### Privacy

Although clients do not directly access the original data of other clients, many inference attacks can recover the original data from model updates and can achieve a quite high accuracy [59]. There are many ways to attack FL, e.g. (i) membership inference [60]: attackers determine whether it is in the training set for a given sample. (ii) Attribute inference [61]: the attacking party determines whether it is involved in training in round t for a given sample attribute. (iii)Feature inference: restore the original data of the target sample by observing the information of the maliciously arranged clients. These attacks can easily compromise the privacy of data providers. Zhu et al. [62] explored gradient deep leakage and experimentally proved that the label matching of images and text obtained can be significantly accurate, they also pointed out that an effective method to circumvent the privacy leakage is gradient pruning. After investigation, we elaborate on the promising FL privacy protection strategies that can be applied to the cloud-edge collaborative architecture as follows:

- *Differential Privacy*: Traditional ML also suffers from privacy leakage, and many privacy-preserving theories have been proposed to safeguard the training, among which differential privacy (DP) is one of the most effective theories [63]. However, DP is harder to deploy and almost ineffective in more complex deep learning tasks, and FL its own does not provide privacy-preserving mechanisms, which popularizes introducing DP into FL. DP works by adding artificial noise e.g. Gaussian noise to the parameters of the clients before aggregating, and different artificial noise brings different levels of privacy protection levels [64]. Wei et al. [65] found that (i) there is a trade-off between model convergence performance and privacy level (ii) fixing the privacy level and increasing the number of clients improves the convergence performance (iii) fixing the privacy level and model convergence, FL has an optimal number of communication rounds. Their work is the basis for applying DP in cloud-edge collaborative architecture, where edge-client and cloud-edge updates need to rationalize the parameters of DP to achieve various trade-offs including the trade-off between communication latency and privacy level. Considering that DP provides a lower level of protection when the scale of clients is relatively small, which is ineffective for cross-silo FL, Triastcyn et al. [66] improved DP by proposing to enhance it with a natural relaxation of DP (BDP). Different from DP, BDP calibrates the noise to the data distribution, and they claim that BDP provides a better level of privacy than DP for the same noise.
- *Homomorphic Encryption*: Homomorphic encryption (HE) and DP share the same goal: to guarantee that the updated gradient can not be deciphered by the attacker when the gradient is leaked. In DP, artificial noise is added to the original data, which may cause data loss problems due to the receiver’s inability to decrypt the noise as well. In contrast, homomorphic secrecy is more secure, allowing direct computation of the encrypted data and only the encrypting party can decrypt the encrypted data. At the same time, HE is more complex than DP, and usually the shortest key length can be tens of times the average gradient length, making the length of the ciphertext unacceptable and leading to extremely inefficient communication. Moreover, current HE usually involves many modular multiplication calculations and large exponential operations, taking up many computing resources originally for local training, which is particularly ineffective for cross-device FL. Considering its security and reliability, in the future, optimized HE may be effective for industrial FL with high privacy requirements. Zhang et al. [67] pointed out that in HE client computation used for HE dominates the training time and exacerbates the communication pressure. They proposed batchcrypt to encrypt gradient in a non-exact manner, and the encryption process is performed on a batch set. Gradient batch processing is actually not simple [68], and most of the generic quantization methods do not support FL; to achieve this, they designed a new quantization scheme. Besides, since this approach causes a loss in the accuracy of the transmitted gradients, they proposed a suitable model pruning algorithm. Batchcrypt greatly improves the training speed of the model (>20%), while significantly reducing the communication (>60%), and after simulations, they claim that batchcrypt also hardly reduces the accuracy of the joint model. Hao et al. [69] combined HE and DP, which theoretically provides higher privacy level. DP is performed by introducing some noise to the raw gradient before uploading them, and then HE is performed, which they claimed can resist FL attacks jointly by edge servers and malicious participants, and can be deployed at a large scale. However, they do not consider the two key pervasive challenges: (i) communication and (ii) computation.

### Personalization

Sometimes the local dataset is too small to train a model with high accuracy, so clients choose to participate in FL with the underlying goal of getting a better model, which costs communication and computation resources, risking a privacy breach at the same time. However there are cases where the quality of the local model may be stronger than the joint model, e.g., a client with a large dataset in cross-silo FL, for which their participation in FL may not be beneficial. Model heterogeneity refers to that different clients may have different requirements for the model. e.g., in a word prediction task, inputting the same “I like ......”, different customers will obviously expect different prediction results. Model heterogeneity issues can be addressed to some extent by applying some personalization methods. We summarize some personalized federation learning (PFL) methods that are applicable to the cloud-edge collaborative architecture as follows:

- *Meta-Learning*: Meta-learning researches how to increase the efficiency of the learning system through experience, which aims at finding approaches to dynamically search for the best learning strategy with the number of tasks increasing [70]. Many researchers have studied applying Meta-learning into FL to enhance the model generalization performance. Jiang et al. [71] discussed the possibility and advantages of *Meta-Learning* in FL and demonstrated that Meta-learning in FL is promising in the future, and they pointed that FL can be seen as a natural application scenario for *Meta-Learning*. Besides, they explain the traditional FedAvg with *Meta-learning* and prove that results derived from fine-tuned FedAvg will be better than merely improving the accuracy of the global model. Fallah et al. [72] studied a personalized variant of FL to find an initial shared model that each client can easily adapt to their local dataset by performing a few steps of gradient descent on their local data. Concretely, they design the meta-functions to replace the global loss function in FedAvg, and the meta-functions also applies to the local training on each client. Chen et al. [73] proposed a federated meta-learning framework FedMeta. A meta-learner rather than a global model is communicated in the framework. Meta-training consists of two phases. The algorithm $\Lambda$ trains a model *f* on the support dataset, and then the model is evaluated on the query dataset where parameters reflecting the training ability of the algorithm will be computed, after that, the parameter $\lambda$ will be updated. There are only two kinds of information communicated: (i) initial model parameters (server to clients) and (ii) test loss (clients to server), which is safer than the naive FL setting, and FedMeta exhibited faster convergence and higher accuracy.
- *Transfer Learning*: Transfer Learning emphasizes the ability of the system to recognize and apply the knowledge and skills learned in previous tasks to new domains or tasks. In the case of insufficient data quality which is pervasive in FL, the introduction of Transfer Learning can better the personalization performance of the model. Under this mechanism, each client can learn their personalized model faster. A group knowledge transfer algorithm, FedGKT, is introduced in [74] which trains a small CNN on resource-constrained devices and transfer local knowledge to a central server periodically. By slightly modifying the existing federated learning structure, Liu et al. [75] proposed Federated Transfer Learning (FTL), which enables the target domain to obtain enough labels from the source domain to build a flexible model. However, it is challenging to implement FTL in practical applications for that too much computation is required. They considered designing a framework combining FTL with HE and secret sharing (SS) for privacy protection, where HE consumes a significant amount of computation while SS avoids the loss of precision with little computation. Chen et al. [76] applied FTL into healthcare and proposed FedHealth for wearable medical devices, which enabled accurate and personalized healthcare suggestions without compromising privacy and security.

## Applications of federated learning in cloud-edge collaborative architecture

In the previous section, we introduced the key technologies of deploying FL in the cloud-edge collaborative architecture, and in this section, we will focus on the applications of FL in the cloud-edge collaborative architecture.

### FL for computation offloading

**Fig. 4:**
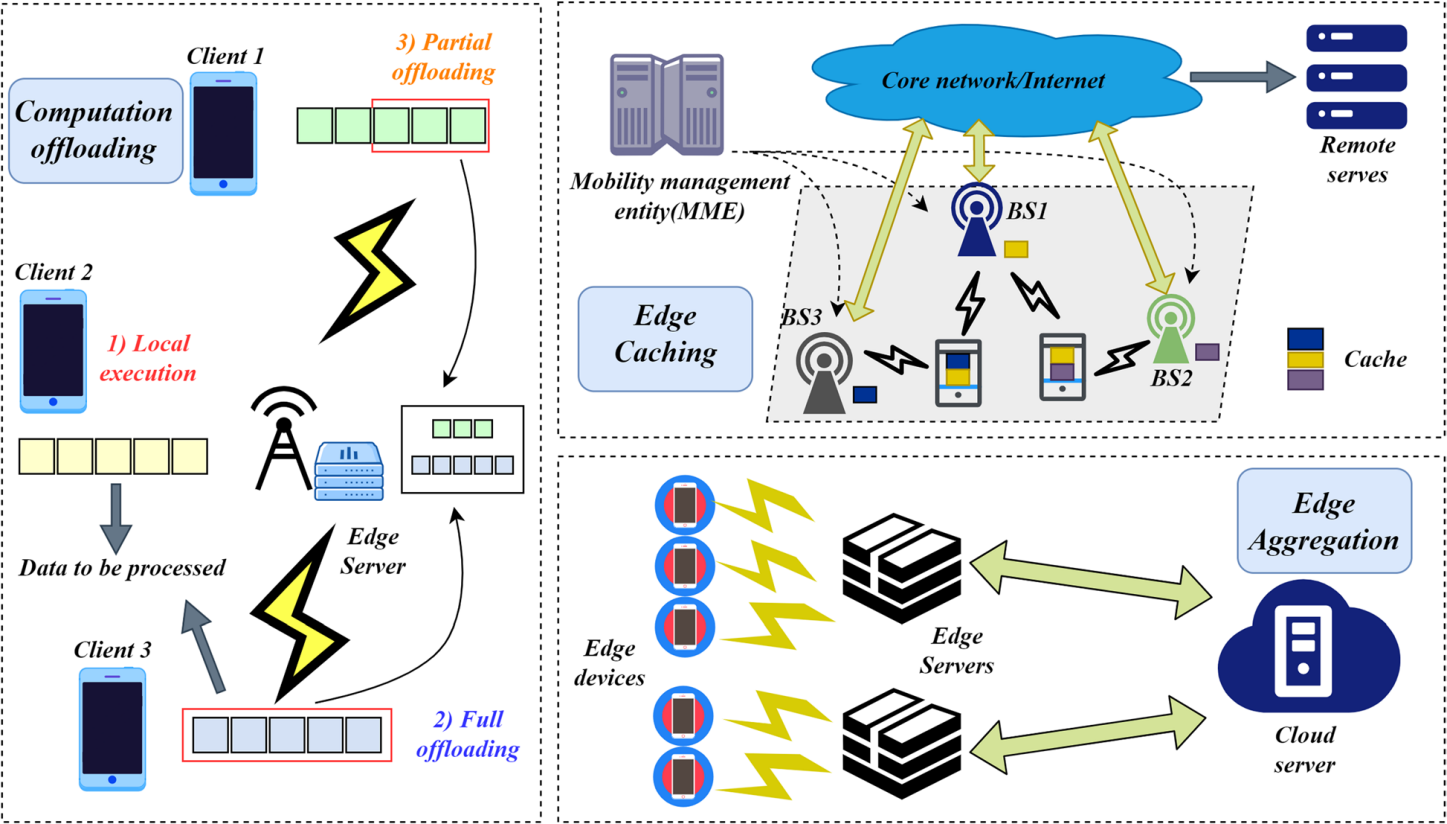
The frameworks of computation offloading, edge caching and edge aggregation

The hardware improvement of mobile devices and the complexity of emerging applications are parallel. However, mobile devices are limited by the battery capacity. In order to extend the battery life of the mobile devices, the computing tasks can be transferred from the mobile devices [77]. However, offloading the tasks to the cloud server brings unsatisfied latency [78], including the communication time between clients and the cloud, as well as cloud processing time. Therefore, offloading the tasks to the edge servers is a better choice, although the execution time of the edge servers is longer than the cloud servers, where deciding what to offload is significant, and the details of computation offloading is demonstrated in Fig. 4, including partial offloading, full offloading and local execution. Some research has employed Deep Reinforce Learning (DRL) to make the offloading decision. Ren et al. [79] considered combining Deep Reinforce Learning and FL to achieve computation offloading. In each client, a task queue is maintained and the tasks are to be offloaded to the edge servers or executed locally. They employed the DRL agent to make the offloading decision and train the agent with FL. Clients download the agent parameters from the edge servers, training locally, and then aggregate local updates through FedAvg. Experiments showed that their FL-based distributed offloading decisions reached centralized methods.

### FL for edge caching

In the cloud-based collaborative architecture, users need to request it from a distant cloud to access the content they expect, which makes the content access slow and takes up precious network resources. In the cloud-edge collaborative architecture, caching content on edge servers (edge caching) can speed up user access and make the communication efficient. However, since the storage resources of edge servers are limited compared to the cloud and cannot cache much content, what to cache is one of the significant problems to be solved in edge caching. Recently, learning-based methods to predict file popularity is proved to be effective, which models user preferences based on the number of service requests, and the type of users, e.g., age, gender, and occupation. However, implementing the scheme will involve users’ privacy and it is insecure to use centralized ML, so there are some researchers considering employing FL to support edge caching. Yu et al. [80] proposed to cache suitable files at the edge servers using a FL-based approach (FPCC). FPCC is a client-edge model, where clients requesting content download encoder models from the server and then train on their local data, where hybrid filtering is used, and local updates are aggregated to the edge servers using FedAvg. Finally, N files are recommended to the edge servers. FPCC outperforms the previous Random, m-$\epsilon$-Greedy, and Thompson Sampling algorithms for file popularity prediction accuracy and provides higher security. Yu et al. [81] considered edge caching in vehicular networks, which is a significant challenge for intelligent transportation. Different from other scenarios, vehicles move fast and the vehicles connected to the edge server tend to change, posing two challenges: (i) The frequent changes of vehicles make the popular files difficult to predict. (ii) The cached contents are easily outdated. To address the above challenges, Yu et al. [81] designed MPCF, an FL-based mobility-aware active caching approach. MPCF utilizes context-aware adversarial autoencoders for prediction, where vehicles receive stored data from RSUs for local training. In addition, they design a strategy of mobility-aware and cache replacement to achieve highly dynamic prediction. They experimentally claim that the dynamic prediction accuracy of MPCF exceeds that of other caching schemes.

### Vehicular networks

Recently, research on edge computing in vehicular networks has been on the rise [82–84]. The goal of vehicular edge computing is to develop computing and communication resources at the edge of the Internet of Vehicles (IoV) and promote artificial intelligence applications in intelligent connected vehicles. However, data leakage may cause massive damage to users and data providers. In addition, resource constraints and dynamic network topology make data privacy protection a challenge.

Ye et al. [85] put forward a selective model aggregation method to guarantee the accuracy and efficiency of FL. Due to the central server being unaware of other details of the vehicle nodes, this setting can protect the private data of the vehicle client. Boualouache et al. [86] used FL to achieve collaborative learning among vehicles while protecting the privacy of vehicles, and finally achieved efficient detection of passive attacks in the Internet of Vehicles. Chen et al. [87] aimed to implement an intrusion detection system in IoV using FL which outperforms existing approaches for common attacks.

### Medicine

Intelligent medical diagnosis based on ML relies on extensive disease samples, however, the disease data stored in each medical center is limited, making it impossible for computers to make accurate diagnoses. Therefore, some research aggregates data from multiple medical centers to a central server for ML and have achieved remarkable success. However, medical data often involves patients’ privacy, and the centralized approach will pose a threat of privacy breach. FL enables the medical data to be stored in each medical center and participate in collaborative training to solve privacy issues.

Due to the significant improvement of wearable and sensor technology, smartphones and wearable devices can collect users’ physiological information and offer important warning of irregular health situations. FL can train large-scale abnormal health detection (AHD) models across participants. However, there are often significant differences between participant data, which existing federated learning methods cannot solve. Guo et al. [88] presented an FL frame FedSens, specifically solving the imbalanced participant data in AHD so that FL can adapt well to AHD. The experiment proves that FedSens is effective. An important significance of federated learning in medical edge computing is to enable remote medical centers lacking advanced diagnostic equipment to obtain more benefits to promote the even distribution of medical resources. In [89–91], FL is applied to COVID-19 diagnosis, Qayyum et al. [89] used cluster-based FL (CFL) to automate COVID-19 diagnosis. While ensuring data security, the performance of the CFL method improved by 16%. Different from [89], Zhang et al. [90] used a novel FL method based on dynamic fusion to determine participating customers according to their local model performance and arranged model fusion according to the client’s training time, enhancing the detection flexibility. Experiments demonstrated that the method outperforms the default setting of FL.

### Cyber security

IoT brings potential applications to many fields such as healthcare, business, smart city. However, due to distributed and heterogeneous characteristics, various attacks such as DDoS and Dos can be quickly introduced to the network. Detecting these attacks and taking measures to defend against them is an important research task.

Huong et al. [92] proposed a new security protocol, Lockedge. They considered that the source of network attacks in areas such as intelligent city monitoring is mainly compromised edge devices, so they deployed attack detection mechanisms at the edge for faster response, and they used lightweight FL to achieve distributed Attack detection to protect data privacy and adapt to resource-constrained terminal devices. Experiments prove that the Lockedge approach outperforms CNN, NN, and RNN methods in accuracy and complexity. Given that a single defender cannot accurately and efficiently detect network attacks, Li et al. [93] proposed using a federated learning method to perform collaborative training on a larger data sample. At 2.7 times the cost, they obtained an accuracy similar to the centralized method. Chen et al. [87] put forward a federated learning-based network intrusion detection algorithm FedAGRU, improving the detection accuracy of poisoning attacks. However, the method requires huge communication costs, so they adopted the attention mechanism to adjust the weight of terminal devices in aggregation, reducing unnecessary local updates. Experiments show that the accuracy of FedAGRU is more robust than that of centralized methods, and the communication efficiency is lower than that of existing federated learning algorithms.

## Challenges and future research directions of deploying federated learning in cloud-edge collaborative architecture

In addition to the above issues, there are still many challenges to the large-scale deployment of federated learning in cloud-edge collaborative architecture, and this section focuses on these challenges, which we summarize in Table 2.

**Table 2:** Applications of Federated Learning in Cloud-Edge Architecture

Refs	Area	Aims	Methods	Dataset	Advantages
Lu et al. [94]	IOV	Protect passenger privacy	GCN	20newsgroups	better than VFL
Ye et al. [85]	IOV	Reduce the Impact of Heterogeneity Problems in Vehicle Clients on Federated Learning Performance	selective polymerization	MNIST & BelgiumTSC	better than approaches based on FedAvg.
Bao et al. [95]	IOV	Implement client selection and networking solutions in a car networking environment	Fuzzy logic algorithm	n/a	Communication-efficient
Boualouache and Engel [86]	IOV	Detect passive mobile attackers in 5G vehicle edge computin	MLP	n/a	Fast Detection & High Accuracy
Xu et al. [96]	IOV	Accurately schedule and dynamically reserve the appropriate amount of multimedia service resources on edge servers.	ST ResNet	n/a	Secure and efficient
Fantacci and Picano [97]	Demand prediction	Protect sensitive user data	FedAvg	MovieLens 1M & MovieLens 100K	better than the approach based on chaos theory and deep learning.
Taïk and Cherkaoui [98]	Household load forecasting	Protect user privacy	FedAvg	n/a	significant gain in the network load
Rahbari et al. [99]	UAV	Improve resource utilization in real-time applications.	Aggregate by scoring weight	n/a	better fairness & energy efficient
Pham et al. [100]	UAV	Improve the transmit power efficiency of UAVs	Decomposition	n/a	Dramatically reduce drone launch power
Chen et al. [101]	Augmented Reality	Improve computational efficiency & Reduce latency	CNN	CIFAR-10	Fewer training iterations
Hsu et al. [102]	Information Security	Android malware detection	SVM	from NICT	Outperforms centralized training systems.
Wang et al. [103]	Industry	Industrial Equipment Troubleshooting	Asynchronous update	n/a	Communication-efficient & Fast Convergence
Zhang et al. [104]	IOMT	Adapt FL to train AHD models	FedSens	real-world AHD applications	strong for biased class distributions
Qayyum et al. [89]	Healthcare	Automated diagnosis of COVID-19	CFL	COVID-19 CT segmentation	Outperforms traditional FL models
Yuan et al. [105]	Intelligent Transport	Traffic flow forecast	FedSTN	n/a	accurate and fast prediction
Vyas et al. [106]	Intelligent Transport	Calculate driving stress and the relationship between driving stress and driving behavior	Long Short-Term Memory Fully Convolutional Network	UAH	High pressure prediction accuracy
Sada et al. [107]	Video Analysis	Distributed video analysis framework	distributed object detection	n/a	Real-time Distributed Object Detection
Chen et al. [87]	Cyber Security	Intrusion detection of wireless networks	FedAGRU	KDD CUP 99 & CICIDS2017	Communication-efficient & strong robustness against poisoning attacks
Li et al. [93]	Cyber Security	Detect network attacks	FLEAM	n/a	Approximate accuracy to centralized training. & Greatly increase detection rate.
Huong et al. [92]	Cyber Security	Quickly and accurately identify cyber attackers	Centralized and distributed methods	BoT-IoT	Accuracy and complexity outperform CNN, SVM and other algorithms.
Hu et al. [108]	Smart City	Urban environment sensing	FRL	n/a	Energy-efficient

### Outlier

In edge federated learning, a group of edge devices is decided for each round of training. The client selection strategy was introduced to personalize the model for each client. However, all these methods implicitly assume that all clients can remain connected to the edge servers. However, edge devices have limited energy, and their network environment is constantly changing, so edge devices are likely to be disconnected during the training process, and these disconnected clients are called outliers. Most of the existing research on dealing with outliers focuses on keeping the training continuing when there are very few outliers, but they cannot cope with many outliers, and the performance of federated learning will be significantly reduced [109]. How to design an edge federated learning setting such that it can maintain stable connections for most clients is a significant challenge.

### Aggregation

Model aggregation is one of the most significant steps in FL, which directly affects the quality of the training. However, in most FL settings, the aggregation is simple and cannot cope with complex cloud-edge collaborative architecture, as Fig. 4. shows. Although people have made some efforts to try to solve (i) Handling of damaged updates [110]. (ii) Avoiding aggressive updates [111]. Further research on robust FL aggregation is still required, e.g. considering mobility aggregation and more secure aggregation.

### Incentive mechanism

In commercial FL, user modeling on clients’ local data can recommend highly accurate content [112], which brings economic benefits. However, the learning process involves users’ private data, and as people pay more attention to their private data, how to design incentive mechanisms to encourage clients to participate in FL is a problem to be solved. Currently, techniques such as game theory [113] and economic theory [114] are applied into the design of incentive mechanisms for FL, but they merely perform well in simulated experiments and are not guaranteed for real environment performance which is dominated by subjectivity.

### Migration

In mobile edge computing, the geographic location of the edge device is uncertain, and the edge device usually establishes a connection with the edge server that is closest to it, which leads to a problem. When another edge server establishes a connection, the server with the newly established connection to the edge device does not have a copy of the previous training, which makes the training impossible to continue, and the network’s performance degrades significantly. How to keep the training going is an important research topic. Much research on service migration in edge computing has been done [115–118]. However, in edge federated learning, the model is relatively large. The delay in transferring model copies between edge servers is significant, which affects the performance of federated learning, so efficient service migration in a cloud-edge collaborative environment is essential to federated learning.

### Asynchronicity

Most of the existing research uses the federated learning setting of synchronous aggregation. However, users have changing power reserves and network quality in an actual network situation. It is difficult for the edge nodes to keep training due to resource constraints, especially in traditional synchronous approaches. Much research has been done to design efficient asynchronous federated learning algorithms in response to this problem.

Chen et al. [119] designed an asynchronous federated learning framework (ASO-Fed). Considering the heterogeneity of edge devices, they required all edge devices to learn online and use asynchronous updates to achieve global aggregation. The results proved that ASO-Fed has a fast convergence rate and satisfactory accuracy. Chen et al. [120] proposed an asynchronous federated learning algorithm to consider local computing and communication resources adapted to the real-world IoT environment. Due to the use of the greedy algorithm, their algorithm is lightweight, and experiments prove that the algorithm is effective. The research on asynchronous federated learning algorithms is currently in infancy. For the purpose of finer simulating the real IoT environment, asynchronous federated learning is a promising method. Notably, most of the current research only deals with the optimization problem of convex loss functions, and future research on non-convex loss functions is necessary to improve adaptability.

### Algorithms

In the edge-cloud collaborative architecture, many researchers are committed to fully leveraging the computing resources at the edge of the network to accelerate federal learning and design more efficient federated learning frameworks, which requires the support of novel efficient algorithms.

- Edge computing algorithms Speeding up model convergence is often for cross-device FL, cross-silo FL usually requires not high on model convergence rates [121]. In cross-device FL, sometimes massive calculations are required to be performed on mobile clients. However, mobile devices are not good at large-scale computation, thus many researchers tried to transfer the computation tasks to the edge servers [79, 122], which is still in the infancy. Some efficient and safe offloading schemes are considered to be successful [123, 124], but they are usually complicated and not suitable for mobile devices. In the future, it may be an important challenge to design lightweight offloading schemes for cross-device FL.
- Federated learning algorithms The famous FedAvg algorithm may not have a good performance in the edge-cloud collaborative architecture [19], especially in Hierarchical federated learning. FedAvg is not necessarily the best choice between Client-Edge and Edge-Cloud. Recently, many frameworks better than FedAvg have been proposed, such as FEDPD [125], fedBN [126]. Besides, conventional FL Algorithms usually employ SGD optimization on both the clients and the server, and some studies have pointed out that using ADAM optimization on the server may get better results [127]. Based on these inspiration, the flexible design of FL algorithms or frameworks may well be an important challenge.

### Split learning

We elaborated on SL in the above sections [3.6]. SL is promising for its high computation efficiency, which is often at the cost of the increase in communication bandwidth. Besides, due to its relay-based training process, the training time may be prolonged. The combination of FL and SL is becoming a research hotspot, researchers try to combine the advantages of FL and SL, most of whom aim at speeding up SL with FL mechanism and keeping the accuracy of SL. Thapa et al. [48] proposed a collaborative learning method combining FL and SL, where after the initialization, the clients perform forward propagation and send the outputs of the cut layer to the central server, then after training on the central server and the clients received the gradients on the smashed data, clients perform backpropagation and lastly, the clients update the model through FedAvg algorithm by the weighted average of the gradients from the clients. Wu et al. [128] proposed the clustered-based method, where they place each of the clients in a cluster, and in each cluster clients perform naive SL, and outside the cluster perform FL which is similar to [48]. Both of their methods are effective and confirm that the combination of FL and SL is promising in the future. However, there are some problems that need to be considered when conducting the combination. (i) One of the biggest novelties of SL is that SL splits the models into several parts and assigns them to different clients for collaborative training. However, how to split the model is one of the challenges, namely model decomposition. Similar to the dynamic aggregation in FL, due to most of the current combination methods only consider the static split process, and the resource availability in the training is not considered carefully, the combination has not reached the ceiling. (ii) When the combination is considered in a larger system, cases can be that different servers are required to be configured differently, the resource allocation can be unreasonable, therefore hierarchical structure can be introduced to make improvements [129]. (iii) Compared to FL, little research has been done on security issues, Guo et al. [130] proved that the hidden vicious can degrade the training and even take control of the whole collaborative training. Thus security and privacy are significant issues to be addressed in the future.

## Conclusion

Federated learning can be well applied to cloud-edge collaborative architecture, in the edge side FL can get access to the extensive edge data generated by end users and preprocess the edge data, and it can be a promising enabling technology for performing learning tasks in the cloud-edge collaborative architecture. In this paper, we elaborate on federated learning and cloud-edge collaborative architecture respectively. Then we summarize the key technologies, applications, and challenges of deploying federated learning in cloud-edge collaborative architecture. In addition to the challenges discussed in this paper, there are many unsolved problems in deploying FL in the novel cloud-edge collaborative architecture. The core motivation of this paper is to guide more people to pay attention to and study FL in the cloud-edge collaborative architecture and provide scientific guidance for future directions.

## Data Availability

Not applicable.
